# Pulp repair response after the use of a dentin-pulp biostimulation membrane (BBio) in primary teeth: study protocol for a randomized clinical trial

**DOI:** 10.1186/s13063-020-04785-2

**Published:** 2020-10-22

**Authors:** Maria Aparecida Andrade Moreira Machado, Tássia Carina Stafuzza, Luciana Lourenço Ribeiro Vitor, Silgia Aparecida da Costa, Sirlene Maria da Costa, Natalino Lourenço Neto, Thais Marchini Oliveira

**Affiliations:** 1 Department of Pediatric Dentistry, Orthodontics and Public Health, School of Dentistry of Bauru, University of São Paulo, Bauru, São Paulo Brazil; 2 Course on Textiles and Fashion, School of Arts, Sciences and Humanities, University of São Paulo, São Paulo, Brazil; 3 Hospital for the Rehabilitation of Craniofacial Anomalies, University of São Paulo, Bauru, São Paulo Brazil

**Keywords:** Vital pulp therapy, Pulpotomy, Chitosan, Tooth, Deciduous, Pulp

## Abstract

**Background:**

Vital pulp therapy aims at maintaining the pulp tissue injured but vital. Thus, the use of capping materials that induce tissue regeneration is a great current trend. This study aims to evaluate clinically and radiographically the pulp repair after the use of dentin-pulp biostimulation membrane in primary teeth.

**Methods:**

Four hundred and sixty-eight teeth from children aged between 5 and 9 years old, both genders, with deep caries lesion with pulp involvement, but no furcal impairment and any sign of necrosis will be selected. The vital pulp therapy will be performed with mineral trioxide aggregate (control group) and dentin-pulp biostimulation chitosan membrane (BBio group). The clinical and radiographic outcomes will be assessed at 12 and 24 months after treatment. The thickness of the dentin barrier will be verified through *Image J2* software. The Wilcoxon signed rank test and Mann-Whitney test will respectively compare the intra- and intergroup clinical and radiographic outcomes. Paired *t* test and independent *t* test will respectively compare the intra- and intergroup radiographic measurements. The logistic regression will be applied, and the degrees of this association will be measured using odds ratio (OR) and 95% confidence interval (95% CI).

**Discussion:**

Therefore, this study protocol aims at new perspectives of vital pulp therapy of primary teeth by employing new easy-handling, low-cost material to keep viable the pulp tissue capable of regenerating and maintain the physiological process of deciduous tooth exfoliation.

**Trial registration:**

Brazilian Registry of Clinical Trials RBR-6vr58b. Registered on 17 February 2019.

## Background

Currently, endodontic treatments focus on reducing the pulp devitalization by either preserving or regenerating the pulp tissue after the pathologic alterations [1–4]. Regenerative procedures use materials to replace the cells or induce the remnant cells to differentiate in new pulp cells [4, 5].

Mineral trioxide aggregate (MTA) is the gold-standard material for clinical procedures of vital pulp therapy of primary teeth because of higher success rates in systematic reviews and meta-analysis [6–10]. Despite its biocompatibility, MTA has a longer setting time, difficult handling, high cost, and a potential for tooth discoloration [9, 11–13].

Technology and innovation aid in developing capping materials tailored for vital pulp repair and regeneration [14–19]. Thus, the search for new bioactive, biodegradable, and nontoxic material is constant [4, 20]. In this scenario and given its commonness regenerative, anti-inflammatory properties, and biocompatibility, chitosan, a natural polymer derivative of chitin, has found several applications in dentistry nowadays [21–23].

Recent studies point out that biochemical and biophysical characteristics of polymers and membranes have great potential for application in different dentistry fields [24–26]. Due the biochemical properties of chitosan and the ability to interact and stimulate pulp cells, chitosan has been exploited in association with other materials to produce new bioactive pulp dressings [23, 27]. Local antimicrobial drug delivery systems may eradicate the pathogenic microbiota or modulate the inflammatory response by decreasing the tissue destruction [4].

After the successful in vitro biophysical and biological characterization of an intraoral multilayer membrane with chitosan [25, 26], this study will aim to verify pulp repair response after the use of a new dentin-pulp biostimulation material in vital pulp therapy of primary teeth. The null hypothesis is that the new dentin-pulp biostimulation material will result in comparable clinical and radiographic success rate of MTA.

## Methods/design

### Study design

In primary teeth, vital pulp therapy goals are the pulp tissue regeneration and the maintenance of the physiological process of deciduous tooth exfoliation. Despite its biocompatibility, MTA has a longer setting time, difficult handling, high cost, and potential for tooth discoloration. Thus, this study aims to evaluate clinically and radiographically the pulp repair after the use of a new dentin-pulp biostimulation chitosan membrane in primary teeth with deep occlusal and occlusoproximal cavities reaching more than 3/4 of the total thickness of carious dentin evaluated by periapical radiograph, with at least two thirds of root length. This will be a double-blinded parallel-group randomized controlled trial composed of two groups: group 1 (G1—control), teeth treated with mineral trioxide aggregate (MTA), and group 2 (G2—experimental), teeth treated with the new dentin-pulp biostimulation chitosan membrane.

Before study group assignment, the allocation concealment will be performed to trial participants in sequentially numbered, sealed envelopes. This closed envelope will include a randomization number. Allocation concealment will be ensured, as the team responsible will not release the randomization number until the patient has been recruited into the trial. The operator will open the envelope and will find the treatment condition to be conducted in the patient. The operator then gives the information about treatment allocation to the patient. The team responsible for recruitment is not allowed to receive information about the group allocation.

One primary tooth per patient will be randomly allocated in each group. If the patient has more than one tooth to include in the study, the tooth closest to the inclusion criteria will be chosen. The allocation criteria will be the cavity type (occlusal and occlusoproximal) and the child’s age. Group allocation will be randomized through computerized stratified sampling (Microsoft Excel®), independently, that is, the patients and legal guardians will remain blinded to intervention group status. Randomization will occur close to the moment of the intervention [18]. Blinding the operator during the allocation sequence and application step will not be possible. The intervention group identifier will be assigned at random and will not be identified during data analysis, that is, the examiners will remain blinded.

### Study setting

Participants will be recruited at municipal schools of Bauru, Sao Paulo, Brazil. The children and legal guardians will be instructed about the research and will read and sign a free and clarified consent term. The treatments will take place in the Pediatric Dentistry Clinics of University of Sao Paulo (Bauru, Sao Paulo, Brazil). Efforts will be made to achieve adherence by explaining the treatment importance. This study will consider the patient-centered, public health, and economic outcomes. Recruitment will take place from December 2019 to August 2020. Each participant will be enrolled in the study for approximately 25 months (1 month for treatment plus 24 months of follow-up), and vital pulp therapy procedures will be performed on week 2 to week 12. This clinical trial follows the guidelines for randomized clinical trials (SPIRIT checklist (Additional file 1)).

### Eligibility: inclusion and exclusion criteria

**Fig. 1:**
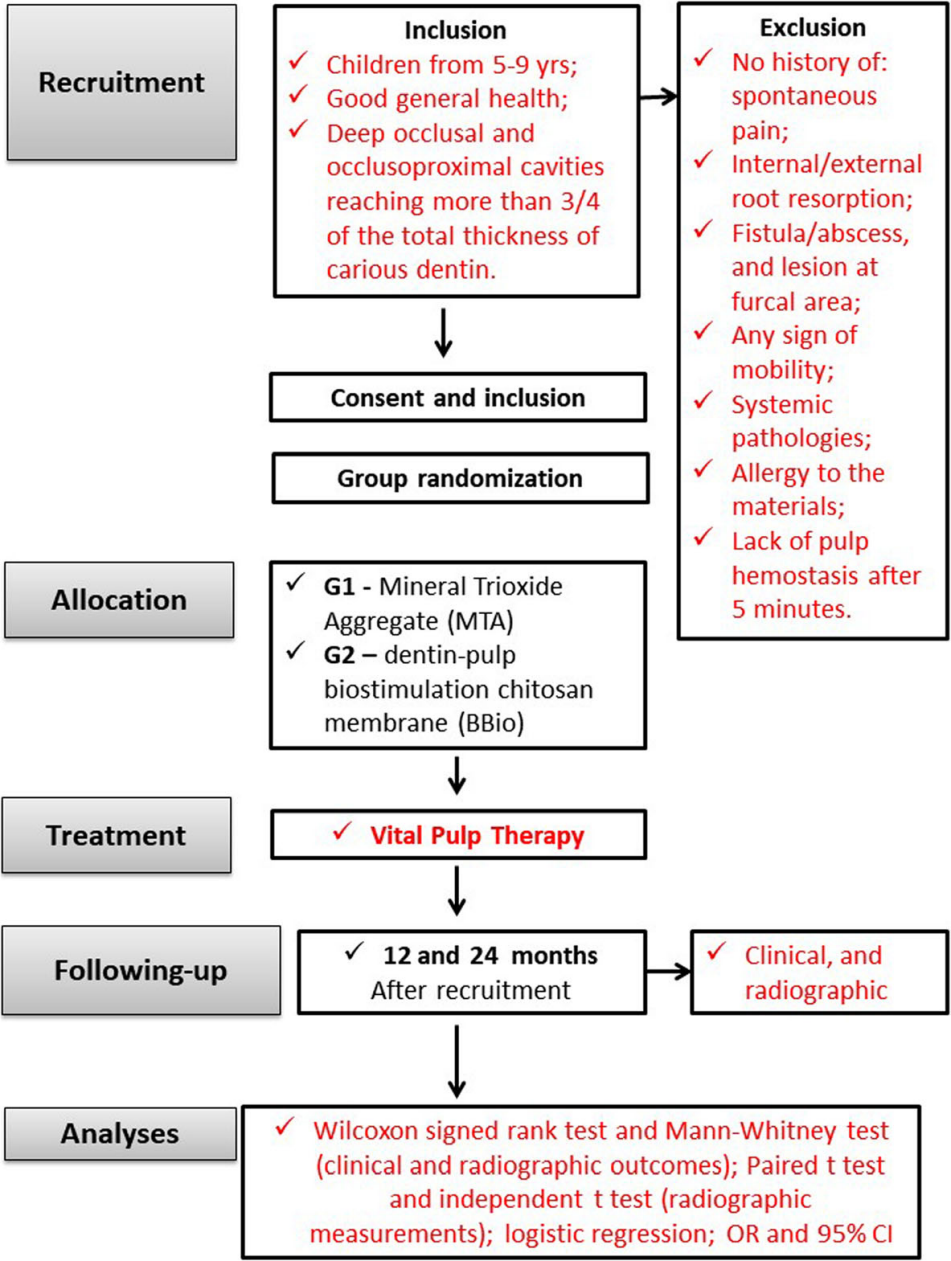
Flowchart of the study

Inclusion criteria will comprise children aged from 5 to 9 years, from both genders, with one restorable maxillary/mandibular first/second primary molar with deep occlusal and occlusoproximal cavities reaching more than 3/4 of the total thickness of carious dentin evaluated by periapical radiograph, with at least two thirds of root length. Exclusion criteria comprised no history of spontaneous pain, internal/external root resorption, fistula/abscess, and lesion at furcal area, and any sign of mobility [1, 2, 28–31]. Also, by the time of clinical procedure, hemostasis will be checked and must occur within 5 min after coronal pulp exposure [28–31]. Exclusion criteria will be the presence of systemic diseases, history of allergic reaction to dental materials, and lack of hemostasis 5 min after coronal pulp exposure (Fig. 1).

### Interventions

#### Clinical procedures

All clinical procedures of this study will be performed by one previously trained and calibrated operator (post-graduate student). A detailed anamnesis and clinical examination will be performed. Next, an intraoral clinical examination with the aid of dental mirror and air jet will screen eligible molars. Then, a periapical radiograph of the eligible primary molars will be taken to verify the furcal conditions. Satisfying all inclusion criteria of the study and after informed consent is given, treatment will be randomly provided in the second visit, according to previously described allocation. The patient and legal guardians will remain blinded regarding the treatment type [32].

After tooth prophylaxis with rubber cup and prophylactic paste (Herjos Tutti Frutt, Vigodent, São Paulo, Brazil), washing, and drying, local anesthesia will be applied (articaine 4% + epinephrine 1:100.000) [28–31]. The cavity preparation will be performed under rubber dam isolation, using water-cooled diamond burs, and caries will be completely removed from the lateral walls and enamel-dentine junction with the aid of low speed round burs (KG Sorensen®, São Paulo, Brazil). The pulp chamber roof will be removed with the aid of round diamond burs (1014-1015) at high speed under copious irrigation. The coronal pulp will be removed with the aid of hand excavators. Pulp vitality will be checked by cut resistance, red color, and hemostasis after 5 min. If necessary, constant irrigation will be performed with sterile saline solution. Pulp stumps will be dried with sterile cotton pellets, and the capping material will be applied according the assigned group [28–31]:

- Control group—MTA (Angelus®, Londrina, Paraná, Brazil) will be mixed on sterile glass plate according to the manufacturer’s instruction (1 powder spoon to 1 drop of distilled water) for 30 s and placed onto the pulp stumps with the aid of spatula and sterile cotton pellet moistened with distilled water. A thin layer of the material will be assured, based on previous studies [14, 30, 31, 33, 34].
- BBio group—the dentin-pulp biostimulation chitosan membranes will be previously prepared in the laboratory with equal parts containing 1.5 g of chitosan (Sigma Aldrich, St. Louis, USA), 1.5 g of alginate, and 1.5 g portland cement-based. Each layer will be individually placed and dried at 30 °C for 15 min. Then, the membrane (composed of three layers) will be dried for more 24 h [25, 26]. After preparation and sterilization, the membrane will be placed on the pulp stumps.

For both groups, a thin layer of liner material (Cimpat white – Septodont, Pomerode, Santa Catarina, Brazil) will be placed onto the study materials, inside the pulp chamber. The final restorations will be performed with resin-modified glass ionomer cement (Vitremer® 3M/ESPE) [28–31]. Just after vital pulp therapy, a periapical radiograph will be taken (immediate postoperative radiograph) [28–30]. The follow-up periods will be 12 and 24 months after treatment [31, 35] (Fig. 1).

#### Radiographic procedures

The child safety will be properly managed by the use of lead apron thyroid collar and ultraspeed x-ray films. The focus/film distance will be approximately 20 cm, assured by universal acrylic positioning stents. A dental x-ray device with 70 kV and 10 Ma will be used. The exposure time will be 0.5 s. All radiographs will be standardized using universal acrylic positioning stents (Han-Shin type). The periapical radiographs will be taken at preoperative, immediate postoperative, and 12 and 24 months of follow-up [30] (Fig. 1).

### Sample size

The minimum sample size calculation was performed based on data from a previous study that found clinical and radiographic success of 94.73% for teeth treated with MTA [14]. For the sample calculation, alpha and beta errors of 5% and 20% will be respectively considered. The sample size also provides 80% power. The sample will be of 143 teeth per group to detect significant differences. A dropout rate of 20% will be estimated, and then, the number of eligible teeth will be 234 per group.

### Statistical methods

All data will be analyzed using the PASW Statistics 21 software (SPSS) (IBM, Armonk, NY, USA). During all study, two trained and calibrated examiners will evaluate the treated teeth clinically and radiographically. Inter- and intra-examiner reliability will be verified by casual and systematic error. The Kolmogorov-Smirnov test will be adopted to test the normality of continuous variables, which will be expressed as median for the variables with a non-normal distribution and a mean value ± SD for the variable with a normal distribution. The Wilcoxon signed rank test will compare the clinical and radiographic outcomes over period. The Mann-Whitney test will compare the clinical and radiographic outcomes between groups. Paired *t* test will be used to compare the radiographic measurements over time. Independent *t* test will compare the radiographic measurements between groups. The level of significance of 5% will be adopted for all comparisons. The logistic regression will be applied, and the degrees of this association will be measured using odds ratio (OR) and 95% confidence interval (95% CI).

### Outcomes

#### Clinical and radiographic evaluations

The teeth will be clinically and radiographically evaluated at 12 and 24 months regarding the pulp response [28–30]. During the follow-up assessment, the following clinical criteria will be verified: spontaneous pain; any signs of mobility, sensitivity to percussion, abscess/fistula, gingival swelling not caused by poor oral hygiene, and restoration failure. The radiographic criteria will include presence/absence of internal/external resorption, signs of furcal impairment, advanced rhizolysis stage, and restoration failure including fracture and loss. Two previously trained and calibrated blinded examiners will perform the clinical and radiographic assessments. Examiners will be previously trained and calibrated in relation to the clinical and radiographic criteria in a pilot study, based on previous studies [30, 31, 33]. Inter- and intra-examiner reliability will be verified by casual and systematic error [36]. The examiners will fill in a form with all data for further analyses of the results.

#### Measurement of the dentin barrier

After the scanning of the radiographs, the images will be analyzed in Image J2 software version 64 bits (National Institutes of Health, NY, USA) [37]. The measurements on the digitized radiographs will be performed at the immediate postoperative period and 12 and 24 months of follow-up. All measurements of the scanned images will be performed by a trained and calibrated examiner blinded to the clinical procedure and the pulp capping materials to perform the measurement of dentin barrier thickness, and points will be marked on the center of the capping material layer (point A) and on the center of the furcal area (point B) [37]. Intra-examiner calibration will be determined by the casual and systematic error [36]. To assure reproducibility, each image will be measured twice at 15-day interval. The mean of these two measurements will be used for the comparisons.

### Data collection methods, management, and monitoring

The clinical and radiographic aspects of teeth will be evaluated at 12 and 24 months after procedures. Data will be collected and registered on case report forms by trained researchers blinded to group allocation. Data quality will be ensured by validation checks that include missing data. Clinical and radiographic data will be inserted directly in clinical and radiographic evaluation forms. The data of the scanned x-rays will be entered in Corel Draw version 13 software for further analysis. Data monitoring committee (DMC) will independently check data and report any problem. DMC is independent from the sponsor and competing interests. The data will be monitored by an independent clinical research assistant, one statistician, and one dental surgeon. No interim analysis beyond that described above is planned.

### Ancillary and post-trial care

After completing the study, participants will continue to receive dental treatments, if needed, in the Pediatric Dentistry Clinics of University of Sao Paulo, Bauru, Sao Paulo, Brazil.

## Discussion

Many pulpectomy and extractions could be avoided whether pulp regeneration therapies would be tailored for primary teeth [4, 38]. Vital pulp therapy is routinely practiced for the treatment of primary teeth focusing on the regeneration of the dentin-pulp complex [4, 17–19, 38]. Thus, the capping material placed on the remaining pulp stumps should have the capacity of inducing the proliferation of stem and angiogenic cells of the pulp to replace the affected ones [38]. In this context, a bioactive material releases calcium ion, induces electroconductivity, produces hydrogen and calcium, and forms an interfacial layer between the material and the dentin with apatite crystals onto the material surface [12, 13].

According to some systematic reviews and meta-analysis, MTA has higher success rates compared to other materials [6–9]. MTA is a bioactive bioceramic endodontic cement, that is, MTA produces hydroxyapatites after hydration and induces a regenerative response by forming nanocrystals [39]. The presence of calcium trisilicate and disilicate accounts for this bioactivity [12, 40, 41]. Despite its biocompatibility, MTA has some drawbacks such as longer setting time, difficult handling, and potential for tooth discoloration [11–13].

To overcome these MTA drawbacks, different materials can be used. The current tissue bioengineering trends aim at replacing the injured cells or stimulating the growth of the injured tissue through biomaterials [4]. Accordingly, future studies should verify the effectiveness of the developing biomaterials [4, 25, 26, 38]. A chitosan-based pulp dressing material seems a wise choice and provides a wise potential once this material exhibits a number of beneficial properties [23, 42]. The chitosan concentrations used to formulate the membranes are biocompatible, safe, and without side effects for humans’ tissues [25, 26]. Also, several biomedical and dental studies employing chitosan as the primary principle of membranes for tissue repair demonstrate an extensively explored field such as tablets, drug delivery systems, and more recently in tissue engineering, showing good results of biocompatibility in contact with human cells [23, 27, 43, 44].

The limitations of the present study include the disadvantages of noncompliance and high dropout rates, and the obstacle to control the period of follow-up. These disadvantages result in investigations with small sample size and the follow-up time. Further studies should be conducted, and the results should be confirmed through longer-term follow-up periods. The measurement of the dentin barrier after procedures of pulp therapy may have some limitations. This method will be used to avoid mistaken measurements due to the difficulty in evaluating the exact thickness of the dentin barrier and the overlapping of the radiographic image of the roots. One other bias of this present study will be the blinding of the operator. Although it is not possible to blind the operator during capping material application, we believe that this will not interfere in the study outcomes. Therefore, this study protocol aims at new perspectives of vital pulp therapy of primary teeth by employing new easy-handling, low-cost materials to keep viable the pulp tissue capable of regenerating and maintain the physiological process of deciduous tooth exfoliation.

## Trial status

This trial, with protocol version 1, began in November 2019 and is still ongoing; patient recruitment is not yet complete and is planned to continue until September 2020. The first patient was included in December 2019, and the study period will end in September 2022.

## Supplementary information

**Additional file 1.** SPIRIT checklist.

## Data Availability

The datasets generated and/or analyzed during the current study will be available from the corresponding author on reasonable request.
